# Analysis of risks of gastric cancer by gastric mucosa among Indonesian ethnic groups

**DOI:** 10.1371/journal.pone.0216670

**Published:** 2019-05-09

**Authors:** Muhammad Miftahussurur, Langgeng Agung Waskito, Ari Fahrial Syam, Iswan Abbas Nusi, I Dewa Nyoman Wibawa, Yudith Annisa Ayu Rezkitha, Gontar Siregar, OK Yulizal, Fardah Akil, Willy Brodus Uwan, David Simanjuntak, Jimmy Bradley Waleleng, Alexander Michael Joseph Saudale, Fauzi Yusuf, Hasan Maulahela, Marselino Richardo, Abdul Rahman, Yoma Sari Namara, Eko Sudarmo, Pangestu Adi, Ummi Maimunah, Poernomo Boedi Setiawan, Kartika Afrida Fauzia, Dalla Doohan, Tomohisa Uchida, Maria Inge Lusida, Yoshio Yamaoka

**Affiliations:** 1 Division of Gastroentero-Hepatology, Department of Internal Medicine, Faculty of Medicine, Universitas Airlangga, Indonesia; 2 Institute of Tropical Diseases, Universitas Airlangga, Indonesia; 3 Department of Environmental and Preventive Medicine, Oita University Faculty of Medicine, Yufu, Japan; 4 Division of Gastroenterology, Department of Internal Medicine, Faculty of Medicine, University of Indonesia, Jakarta, Indonesia; 5 Division of Gastroentero-hepatology, Department of Internal Medicine, Faculty of Medicine University of Udayana, Denpasar, Indonesia; 6 Faculty of Medicine, University of Muhammadiyah Surabaya, Surabaya, Indonesia; 7 Division of Gastroentero-Hepatology, Department of Internal Medicine, Faculty of Medicine, University of Sumatera Utara, Medan, Indonesia; 8 Center of Gastroentero-Hepatology, Department of Internal Medicine, Faculty of Medicine, Hasanuddin University, Makassar, Indonesia; 9 Department of Internal Medicine, Santo Antonius Hospital, Pontianak, Indonesia; 10 Department of Internal Medicine, Yowari Hospital, Jayapura, Indonesia; 11 Division of Gastroentero-hepatology, Department of Internal Medicine, Faculty of Medicine, University of Sam Ratulangi, Prof. Dr. RD Kandou Hospital, Manado, Indonesia; 12 Department of Internal Medicine, Prof. Dr. W. Z. Johannes General Hospital, Kupang, Indonesia; 13 Division of Gastroenterohepatology, Department of Internal Medicine, Dr. Zainoel Abidin General Hospital, Banda Aceh, Indonesia; 14 Department of Internal Medicine, Merauke City General Hospital, Merauke, Indonesia; 15 Department of Internal Medicine, Kolaka General Hospital, Kolaka, Indonesia; 16 Department of Internal Medicine, Anutapura General Hospital, Palu, Indonesia; 17 Department of Internal Medicine, Dr. Hasan Busori General hospital, Ternate, Indonesia; 18 Department of Molecular Pathology, Oita University Faculty of Medicine, Hasama-machi, Yufu-City, Oita, Japan; 19 Department of Medicine, Gastroenterology and Hepatology Section, Baylor College of Medicine, Houston, Texas, United States of America; 20 Global Oita Medical Advanced Research Center for Health, Hasama-machi, Yufu-City, Oita, Japan; Istituto di Ricovero e Cura a Carattere Scientifico Centro di Riferimento Oncologico della Basilicata, ITALY

## Abstract

Indonesia is a big country with multiethnic populations whose gastric cancer risks have not been elucidated. We performed a nationwide survey and obtained histological specimens from 1053 individuals in 19 cities across the country. We examined the gastric mucosa, the topography, the atrophic gastritis risk factors, and the gastric cancer risk scores. Almost half (46.1%) of the patients with dyspeptic symptoms had histological abnormalities; chronic (36.3%) and atrophic gastritis (28.9%) being the most frequent. Individuals of the Timor ethnicity had the highest prevalence of acute (52.6%) and chronic gastritis (68.4%), even those negative for *H*. *pylori*. Our topographic analysis showed the majority of patients had predominantly antral acute and chronic gastritis. A multivariate logistic regression model showed age (Odds ratio [OR], 1.107), Timor ethnicity (OR, 8.531), and *H*. *pylori* infection (OR, 22.643) as independent risk factors for presence of atrophic gastritis. In addition, the gastric cancer risk score was highest in those from Timor, Papuan, and Bugis ethnic populations. Overall, Indonesia is a low-risk gastric cancer country. However, several ethnic groups displayed severe gastric mucosa symptoms suggesting policy makers should focus on those ethnic groups to perform gastric cancer screenings and to eradicate *H*. *pylori*.

## Introduction

Severe atrophic gastritis (AG) with intestinal metaplasia (IM) is an initial marker for gastric cancer [1, 2], the world’s fifth most common cancer and the third leading cause of cancer death based on report from Global Cancer Incidence, Mortality and Prevalence (GLOBOCAN) 2018. (http://gco.iarc.fr/). AG is an inflammatory reaction characterized by the loss of gastric glandular structures, which are replaced by connective tissue (non-metaplastic atrophy) or by inappropriate glandular structures, (metaplastic atrophy) [3]. The current gastric carcinogenesis model states that AG leads to IM, resulting in gastric gland morphological changes, and it will lead to gastric cancer progression [4]. Severe AG is mostly associated with *Helicobacter pylori* infection which increasing gastric pH and allow for abnormal bacterial overgrowth in the stomach, which in turn leads to abnormal local metabolism of dietary constituents such as nitrates that can produce carcinogens locally [5]. The guidelines recommend that individuals with severe AG and IM be kept under surveillance [6].

In addition, infiltration of lymphocytes/plasma cells and neutrophils predominating in the corpus, and IM in the antrum and/or corpus, have been closely associated with gastric cancer [7]. However, not only the degree of AG, but also its distribution is important [8]. In high-risk gastric cancer countries, the corpus-predominant active gastritis is the typical form, while the antrum-predominant gastritis has a high prevalence in the countries with low risk of gastric cancer [9]. In general, the risk of gastric cancer decreases in order from pan-gastritis with corpus atrophy, followed by corpus-predominant gastritis, pan-gastritis without corpus atrophy, and antrum-predominant gastritis [8]. Other risk factors associated with gastric cancer such as age [10], smoking [11], and alcohol habits [12] have also been reported. A modified gastric cancer risk index (GCRI) [13] was developed based on the criteria initially proposed by Meining et. al [7, 13], which showed a benefit as a detection tool. The score of modified GCRI was assessed according to the conventional histological evaluation score, including IM, gastritis distribution, and AG. The addition of AG in the assessment of modified GCRI was due to the common prevalence of AG in the high risk gastric cancer countries [13]. In addition, the operative linked gastritis atrophy (OLGA) score has been shown to be a good marker for gastric cancer risk even in very heterogenic populations [14].

Indonesia is a multi-ethnicity country with more than 13,600 islands and 300 ethnic groups with a total area of about 5,120 kilometers (3,181 mi) from east to west and 1,760 kilometers (1,094 mi) from north to south. Generally, the prevalence of *H*. *pylori* infection has been shown to be low (10.4%) [15–18]. However, we recently confirmed much higher risks of being infected with *H*. *pylori* in individuals of particular ethnic groups, such as Papuan, Bataknese, and Bugis than the risks for individuals in the Javanese population [16]. In addition, the predominant type of *H*. *pylori* strain in Indonesia is a virulent type (e.g., East-Asian type CagA, *vacA* s1-m1, and *oipA* ‘on’) [19]. Although Indonesia is classified as a low-risk gastric cancer with an age-standardization-rate (ASR) of 2.8/100,000 population (GLOBOCAN, 2012), the area is very wide and comprises multiethnic populations, and we hypothesized that some ethnic groups may have a higher risk of gastric cancer than others. To our knowledge, the risk to develop gastric cancer based on the gastric mucosal status in Indonesia has not been studied. Indonesia is an ideal country for comparison of gastric mucosal status and gastric cancer risk analysis among populations with low and high prevalence of *H*. *pylori*. In particular, it is an ideal place to analyze *H*. *pylori*-negative gastritis and its association with risk factors. To increase the diagnosis validity of *H*. *pylori*-negativity, we used immunohistochemistry to confirm *H*. *pylori* infection by histopathology. For this study we investigated and recorded the presence, type, and pattern of gastritis using samples obtained from individuals in several regions of Indonesia in a nationwide study. We also examined the prevalence and risk factors associated with *H*. *pylori*-negative gastritis. Moreover, we drew a gastric cancer risk map considering several ethnic groups in Indonesia.

## Materials and methods

### Study population

**Fig 1:**
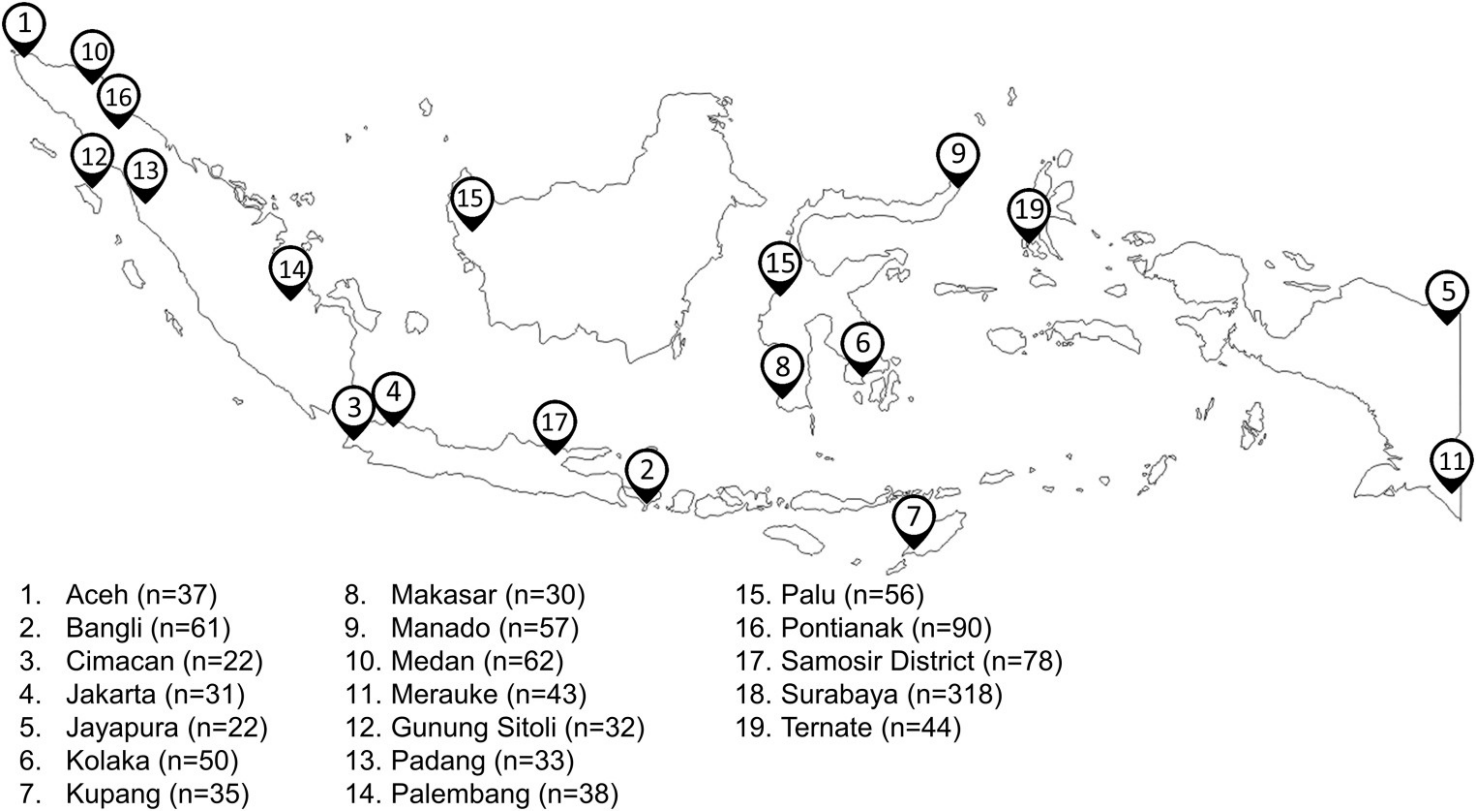
Cities where we performed endoscopy examinations across Indonesian Islands. The asterisk symbol indicates the enrolled patients from our previous publications [15, 16, 19, 20]. The individuals enrolled in our previous study [15, 16, 19, 20] from Samosir District and Surabaya were 31 and 296, respectively.

We conducted a cross-sectional study and performed a nationwide endoscopic survey to 1,236 adult dyspeptic patients in 19 cities across Indonesia from August 2012 to March 2017, including cities from the five largest islands (Fig 1). We included all patients with dyspepsia symptom. This number of endoscopic patients also included 849 patients from our previous studies which was not used for the same analysis [15, 16, 19, 20]. However, among those 849 patients, we could not obtain biopsy specimens from 97 patients in Malang; thus, in the end, we enrolled 1,139 patients in this study. We examined the 387 new recruited patients with dyspepsia from Cimacan (22), Kolaka (50), Merauke (43), Gunung Sitoli (32), Padang (33), Palembang (38), Palu (56), Samosir District (47), Surabaya (22), and Ternate (44). Prior to the endoscopic examination, we interviewed each patient and obtained socio-demographic data, including smoking and alcohol drinking habits, the usage of NSAIDs, PPIs or H_2_-blockerd, the history of *H*. *pylori* eradication therapy, and medical history related to gastroduodenal diseases. We excluded 101 patients, consisted of 18 disagreed to participate patients, 49 were bleeding related to esophageal varices, 21 patients with history of partial/total gastrectomy and 13 patients with previous *H*. *pylori* eradication. We obtained written informed consents from all participants, and the Institutional Review Boards or the Ethics Committees of Dr. Cipto Mangunkusumo Teaching Hospital (Jakarta, Indonesia), Dr. Soetomo Teaching Hospital (Surabaya, Indonesia), Dr. Wahidin Sudirohusodo Teaching Hospital (Makassar, Indonesia), and Oita University Faculty of Medicine (Yufu, Japan) approved the study protocol. We obtained one gastric biopsy from the lesser curvature of the antrum, approximately 3 cm from the pyloric ring and another one from the greater curvature of the corpus, approximately 8 cm from esophageal-cardia junction. We used those gastric biopsies for histological diagnoses. Expert endoscopists performed all of the gastric biopsy procedures. We diagnosed peptic ulcer diseases according to endoscopic findings and gastritis by histological examinations.

### Histology and immunohistochemistry

All biopsy materials for histological testing were fixed in 10% buffered formalin and embedded in paraffin. Serial sections were stained with haematoxylin and eosin and May-Giemsa stain. The degree of neutrophil activity, inflammation, atrophy, IM, and bacterial density were classified into four grades according to the updated Sydney system (0, normal; 1, mild; 2, moderate; and 3, marked) [21]. Samples with bacterial loads ≥grade 1 were considered positive for *H*. *pylori*.

We considered patients as presenting acute and chronic gastritis when if we observed activity based on the neutrophil infiltration and inflammation (indicated by monocyte infiltration scores equal to or greater than 1). We regarded patients as having AG if they presented atrophy in either/both locations regardless of the presence of either monocyte or neutrophil infiltrations. As for the presence of IM in either/both locations we considered the patient as having IM regardless of other gastritis findings. In addition, we also classified acute and chronic gastritis based on the topographical distribution (antral-predominant gastritis if the neutrophil and monocyte infiltration scores in the antrum were greater than those in the corpus; corpus-predominant gastritis if the neutrophil and monocyte infiltration scores in the antrum were lower than those in the corpus; and pan-gastritis if the neutrophil and monocyte infiltrations were equal between the antrum and corpus) [22–24]. In addition, we assessed the AG stages based on topographic locations (antrum and corpus) and according to the Operative Link on Gastritis Assessment (OLGA) system [25]. GCRI scores were assessed according to the classification developed by Tanaka et al [13], assessing five variables, including atrophy scores both in the antrum and corpus, the presence of IM both in the antrum and corpus, and the distribution of chronic gastritis.

To increase the accuracy for detecting *H*. *pylori*, we performed immunohistochemical confirmations, as described [26]. Briefly, after antigen retrieval and inactivation of endogenous peroxidase activity, we incubated tissue sections with anti-α-*H*. *pylori* antibody (DAKO, Glostrup, Denmark) overnight at 4°C. After washing, the sections we incubated them with biotinylated goat anti-rabbit IgG (Nichirei, Tokyo, Japan), followed by incubation with an avidin-conjugated horseradish peroxidase solution (Vectastain Elite ABC Kit; Vector Laboratories, Burlingame, CA, USA). We detected peroxidase activity by using an H_2_O_2_/diaminobenzidine substrate solution. To minimize potential bias, the same experienced pathologist (TU) who also performed experiments for Myanmar, Vietnam, Bhutan, Dominican Republic, and Indonesia [18, 27–31] evaluated all the specimens in this study.

### Statistical analysis

We tested discrete variables using the Chi-square test. The association of GCRI and the ethnic group was tested using Mann–Whitney *U* test. We calculated the odd ratio (OR) of atrophic occurrence using a binary logistic regression model, whereas the OR of the OLGA score severity, which is categorized as an ordinal variable, was calculated using an ordinal regression model. A P value ≤0.05 was considered statistically significant. We carried out all calculations using the SPSS software, version 23 (SPSS, Chicago, IL, USA).

## Results

Among 1,139 examined patient specimens, the histological specimens from 86 patients got dried during transportation to Japan (where the histological diagnoses were to be performed) resulting in poor quality for histology examination. Hence, we did not use those specimens in our analyses; in total, we analyzed 1,053 histology specimens obtained from Banda Aceh (38 specimens), Medan (43), Samosir District (56), Padang (33), and Palembang (38) in Sumatera Island; Gunung Sitoli (32) in Nias Island; Jakarta (31), Cimacan (21), and Surabaya (302) in Java Island; Pontianak (67) in Kalimantan Island; Manado (57), Makassar (30), Palu (55), and Kolaka (50) in Sulawesi Island, Ternate (44) in Ternate Island; Jayapura (21) and Merauke (43) in Papua Island; Kupang (33) in Timor Island; and Bangli (59) in Bali Island. The specimens were collected from 596 men and 457 women with a mean age of 46.25 ± 13.7 years (range, 14–83 years). We found significant differences in the mean age of individuals depending on their city of residence (P < 0.001) with patients from Banda Aceh being the youngest (37.3 ± 13.2 years) and those from Medan the oldest (51.37 ± 13.9 years). According to the endoscopic examination, 975 patients were diagnosed as having gastritis, 77 patients as having peptic ulcer disease, and 1 patient as having gastric cancer. In total, patients with peptic ulcer disease were significantly older than those with gastritis (51.9 vs. 45.9, P = 0.01), with no significant mean age difference by gender. Based on our histological examination, we identified 485 patients (43.4%) with abnormal histologic results (at least one score of monocyte infiltration (inflammation), neutrophil infiltration (activity), atrophy, or IM).

### Acute gastritis

Among 1,053 patients, 178 (16.9%) presented acute gastritis (Table 1). Men had higher acute gastritis prevalence than women, but the difference was not statistically significant. Patients with *H*. *pylori-positivity* had significantly a higher acute gastritis prevalence than patients negative for *H*. *pylori* (100/106, 94.3% vs. 78/947, 8.2%; P < 0.001). In addition, *H*. *pylori* infected patients had 185 fold of odds to have acute gastritis than non infected patients (95%CI = 78.9–436.9). Patients in the Timor ethnic group had the highest acute gastritis prevalence, followed by those in the Bataknese and Papuan ethnicities (52.6%, 31.3%, and 26.1%, respectively) regardless the *H*. *pylori* infection status. We found an association between acute gastritis prevalence and the ethnic groups (P < 0.001). After adjusting for age and gender; Timor, Bataknese, and Papuan ethnics still had the highest prevalence of acute gastritis compared to Aceh ethnics (OR, 22.49, 95%CI = 5.95–84.92, P < 0.001; OR, 8.60, 95%CI = 2.49–19.44, P < 0.001; and OR, 8.56, 95%CI = 2.36–31.07, P = 0.001, respectively). In our analysis of only the 947 *H*. *pylori-*negative patients, we found those in the Timor ethnic still have the highest prevalence of acute gastritis (7/25, 28.0%), followed by those in Dayak and Nias ethnic (7/45, 15.5% and 4/32, 12.5%, P = 0.01, respectively, S1 Table). Antral-predominant acute gastritis was the most frequent presentation before other locations like the corpus-predominant and pan-gastritis (148/178, 76.4% vs. 17/178, 9.5%; and 25/178, 14.0%, P < 0.001, Table 2). All Ternatese, Minahasanese, and Kaili ethnic groups had antral-predominant acute gastritis. We saw a similar trend in Papuan, Bataknese and Balinese populations (15/17, 88.2%; 27/32, 84.4%, and 10/12, 83.3%, respectively). Overall, we found the antral-predominant status was still more frequent than corpus-predominant and pan-gastritis even among the *H*. *pylori-*negative patient group (55/78, 70.5%; 10/78, 12.9%; and 13/78, 16.7%, respectively, P = 0.238).

**Table 1:** The overall observation of gastric mucosal condition in Indonesia.

Characteristic	n	Type of Gastric Abnormalities (%)				
		All Type	Acute Gastritis	Chronic Gastritis	Atrophy Gastritis	Intestinal Metaplasia
**Overall**	1053	485 (46.1)	178 (16.9)	350 (36.3)	305 (28.9)	30 (2.8)
**Gender**						
Men	596	272 (45.3)	98 (16.4)	188 (31.5)	177 (29.7)	18 (3.1)
Women	457	213 (46.6)	80 (14.4)	162 (33.9)	128 (28.1)	12 (2.6)
***H*. *pylori***						
Positive	106	106 (100)	100 (94.3)	105 (99.1)	92 (86.7)	13 (12.3)
Negative	947	379 (40.1)	78 (8.2)	245 (25.8)	213 (22.5)	17 (1.8)
**Ethnic Group**						
Aceh	73	20 (27.4)	3 (4.1)	12 (16.4)	7 (9.5)	1 (1.4)
Balinese	62	28 (45.1)	12 (19.3)	22 (35.4)	20 (32.3)	1 (1.6)
Bataknese	102	59 (57.8)	32 (31.3)	49 (48.0)	45 (44.1)	6 (5.9)
Bugis	99	52 (52.5)	21 (21.2)	39 (39.4)	35 (35.4)	4 (4.1)
Chinese	128	61 (47.7)	17 (13.2)	48 (37.5)	30 (23.4)	1 (0.8)
Dayak	47	27 (57.4)	9 (19.1)	19 (40.4)	15 (31.9)	1 (2.1)
Javanese	233	107 (45.9)	23 (9.9)	78 (33.4)	49 (21.0)	4 (1.7)
Ternatese	47	12 (25.5)	4 (8.5)	4 (8.5)	12 (25.5)	1 (2.1)
Malay	37	8 (21.6)	5 (13.5)	3 (8.1)	5 (13.5)	1 (2.7)
Minahasa	53	23 (43.3)	7 (13.2)	14 (26.4)	20 (37.7)	5 (9.4)
Nias	33	10 (30.3)	5 (15.1)	7 (21.2)	7 (21.2)	1 (3.1)
Kaili	12	3 (25.0)	1 (8.3)	2 (16.7)	1 (8.3)	0 (0.0)
Papuan	65	39 (60.0)	17 (26.1)	24 (36.9)	33 (50.7)	2 (3.1)
Timor	38	31 (81.6)	20 (52.6)	26 (68.4)	24 (63.15)	2 (5.3)
Tolaki	24	5 (20.8)	2 (8.3)	3 (12.5)	2 (8.3)	0 (0.0)

Abbreviations: AcG, Active Gastritis; CG, Chronic Gastritis; AG, Atrophic Gastritis; IM, Intestinal Metaplasia.

**Table 2:** Topography of acute gastritis in Indonesia.

Characteristic	n	Predominant Location (%)		
		Antral	Corpus	Pan-gastritis
**Total**	178	148 (76.4)	17 (9.5)	25 (14.0)
Men	98	77 (78.6)	9 (9.2)	12 (12.2)
Women	80	59 (73.8)	8 (10.0)	13 (16.3)
**Ethnic Group**				
Aceh	3	3 (100)	0 (0.0)	0 (0.0)
Balinese	12	10 (83.3)	1 (8.3)	1 (8.3)
Batak	32	27 (84.4)	2 (6.3)	3 (9.4)
Bugis	21	13 (61.9)	2 (9.6)	6 (28.6)
Chinese	17	13 (76.5)	3 (17.6)	1 (5.9)
Dayak	9	7 (77.8)	1 (11.1)	1 (11.1)
Javanese	23	15 (65.2)	4 (17.4)	4 (17.4)
Ternatese	4	4 (100)	0 (0)	0 (0.0)
Malay	5	3 (60.0)	1 (20.0)	1 (20.0)
Minahasanese	7	7 (100)	0 (0)	0 (0.0)
Nias	5	4 (80)	0 (0)	1 (20.0)
Kaili	1	1 (100)	0 (0.0)	0 (0.0)
Papuan	17	15 (88.2)	1 (5.9)	1 (5.9)
Timor	20	14 (70.0)	1 (5.0)	5 (25.0)
Tolaki	2	0 (0.0)	1 (50)	1 (50.0)
***H*. *pylori* status**				
Positive	100	81 (81.0)	7 (7.0)	12 (12.0)
Negative	78	55 (70.5)	10 (12.9)	13 (16.7)

### Chronic gastritis

Overall, we found 350 patients (36.3%) with chronic gastritis, more than the number of patients with acute gastritis. In contrast, women had a higher prevalence of chronic gastritis than men (188, 31.5% vs. 162, 33.9%, Table 1). As we had predicted, *H*. *pylori-*positive patients had a higher odds for presence of chronic gastritis than *H*. *pylori-*negative patients (OR, 300.85 [95%CI, 41.76–2,167.23], P < 0.001). In the case of acute gastritis, Timor and Bataknese ethnics had the highest prevalence of chronic gastritis in Indonesia (26/38, 68.4% and 49/102, 48.0%, respectively), followed by Dayak, Bugis, and Chinese ethnics (19/47, 40.4%; 39/99, 39.4%; 48/128, 37.5%, respectively; P < 0.001, Table 1). After adjustment for with gender and age, Timor, Bataknese, Dayak, Bugis, Papuan, Chinese and Balinese ethnicities had higher risks to develop chronic gastritis than Malay ethnicities (OR, 22.49, 95%CI = 5.95–91.81; OR, 9.58, 95%CI = 2.75–33.33; OR, 8.03, 95%CI = 2.14–30.10; OR, 7.22, 95%CI = 2.06–25.23; OR, 7.12, 95%CI = 1.96–25.81; OR, 6.33, 95%CI = 1.83–21.80; and OR, 6.24, 95%CI = 1.71–22.75; respectively, all P < 0.05). We did not find any association between usage of proton-ump inhibitors (PPIs) and the prevalence of chronic gastritis (P = 0.851). After defining severe chronic gastritis as an inflammation with scores ≥2 regardless the biopsy location, Timor and Dayak ethnicities were the two populations with the highest severe chronic gastritis prevalence, although we found no significant association between the presence of severe chronic gastritis prevalence and ethnic groups (5/26, 19.23% and 3/19, 15.79%, respectively, P = 0.29, S2 Table). Patients with acute gastritis had mostly antral-predominant gastritis; but, those with chronic pan-gastritis were more (63/350, 18.0%) than those with corpus-predominant gastritis (20/350, 5.7%), with individuals in the Bugis ethnicity presenting the highest prevalence (Table 3). *H*. *pylori* infection did not change the distribution of predominant topography of chronic gastritis (P = 0.192).

**Table 3:** Topography of chronic gastritis in Indonesian patients.

Characteristic	n	Predominant Location (%)		
		Antral	Corpus	Pan-Gastritis
**Total**	350	267 (76.3)	20 (5.7)	63 (18.0)
Men	188	141 (75.0)	10 (5.3)	37 (19.7)
Women	162	126 (77.8)	10 (6.2)	26 (16.1)
**Ethnic Group**				
Aceh	12	10 (83.3)	0 (0.0)	2 (16.7)
Balinese	22	19 (86.4)	1 (4.5)	2 (9.1)
Batak	49	41 (83.7)	3 (6.1)	5 (10.2)
Bugis	39	24 (61.5)	2 (5.1)	13 (33.3)
Chinese	48	38 (79.2)	4 (8.3)	6 (12.5)
Dayak	19	16 (84.2)	1 (5.3)	2 (10.5)
Javanese	78	54 (69.2)	4 (5.1)	20 (25.6)
Maluku	4	3 (75.0)	0 (0.0)	1 (25.0)
Malay	3	1 (33.3)	1 (33.3)	1 (33.3)
Minahasa	14	13 (92.8)	1 (7.1)	0 (0.0)
Nias	7	5 (71.4)	0 (0.0)	2 (28.6)
Palu	2	2 (100)	0 (0.0)	0 (0.0)
Papuan	24	21 (87.5)	1 (4.17)	2 (8.3)
Timor	26	19 (73.1)	1 (3.8)	6 (23.1)
Tolaki	3	1 (33.3)	1 (33.3)	1 (33.3)
***H*. *pylori* status**				
Positive	105	85 (80.9)	7 (6.7)	13 (12.4)
Negative	245	182 (74.3)	13 (5.3)	50 (20.4)

We classified chronic acute gastritis as a subgroup of chronic gastritis with the presence of neutrophil infiltration ≥1 in either the antrum or the corpus, as described previously [32]. We found that almost half (46.8%, 164/350) of the cases with chronic gastritis had neutrophil infiltration. *H*. *pylori*-infected patients had a significantly higher prevalence of neutrophil infiltration than non-infected individuals (99/105, 94.3% vs. 65/245, 26.5%, OR = 45.69, 95%CI = 19.11–109.22, P < 0.001). We also found a significantly higher AG prevalence among the individuals with acute chronic gastritis than that in patients with non-chronic acute gastritis (126/164 [76.8%] vs. 54/186 [40.9%], OR = 8.28, 95%CI = 5.00–13.11, P < 0.001), but we found no difference in the IM prevalence between patients with acute chronic gastritis and those with non-acute chronic gastritis (12/186 [6.5%] vs. 12/164 [7.3%], P = 0.914) (S3 Table).

After analyzing only the 947 *H*. *pylori-*negative patients, patients from Timor were still the most frequent (13/25, 52.0%) of chronic gastritis, followed by the Chinese and Bugis ethnic groups (41/121, 37.78% and 23/83, 33.8%, P < 0.001, respectively). Antral-predominance was also still the most frequent gastritis type (182/245, 74.3%). Interestingly, the percentage of chronic acute gastritis decreased to 26.53% (65/245) in *H*. *pylori-*negative patients and it was significantly lower than that in the overall study population (P < 0.001). Among *H*. *pylori*-negative patients, we also observed an association between chronic active gastritis and AG but no association between AG and chronic gastritis (40/65 [61.54%] vs. 49/180 [27.22%], OR = 4.27, 95%CI = 2.35–7.77, P < 0.001), or between AG with IM (2/65[3.07%] vs. 10/180[5.55%], P = 0.646).

### *H*. *pylori*-negative gastritis

Among all *H*. *pylori-*negative patients, we classified those without gastritis (non-gastritis) as those with absence of neutrophil and mononuclear cells, as described [33]. We categorized the majority of patients as being *H*. *pylori*-negative non-gastritis cases (72.7%, 689/947). We observed that Timor, Dayak, Javanese, Chinese, Bataknese, Bugis, Balinese, Nias, and Banda Aceh ethnic groups had significantly higher risks for presence of gastritis even in the absence of *H*. *pylori* than patients in Ternatese (Table 4). Also, after changing the reference for the logistic regression model to Malay and Tolaki ethnicities; Timor, Dayak, Javanese, Chinese, and Bataknese ethnic groups still displayed significantly higher risks for presence of gastritis in the absence of *H*. *pylori* (S4 Table). We found no association between smoking and alcohol consumption habits and the prevalence of *H*. *pylori*-negative gastritis (P = 0.656 and P = 0.395, respectively). When we excluded the patients with IM and AG, we observed, among the 724 remaining patients, that those in the Dayak, Timor and Javanese ethnic groups had higher odds for presence of gastritis in the absence of *H*. *pylori* than those in the Malay ethnic group (OR, 8.70, 95%CI = 1.75–43.16; OR 8.29, 95%CI = 1.25–54.70; OR, 5.98, 95%CI = 1.37–25.99; all P < 0.05, respectively).

**Table 4:** *H*. *pylori*-negative gastritis in Indonesia.

Characteristic	n	Case (%)	Crude OR	95% CI	P Value
**Gender**					
Men	534	132 (24.7)	1.00	-	-
Women	413	126 (30.5)	1.34	1.003–1.782	0.048
**Age Group**					
≤18 years	5	3 (60.0)	7.03	1.098–44.977	0.040
18–29 years	108	19 (17.6)	1.00	-	-
30–39 years	216	50 (23.1)	1.41	0.784–2.539	0.251
40–49 years	229	62 (27.1)	1.74	0.979–3.090	0.059
50–59 years	216	61 (28.2)	1.84	1.035–3.283	0.038
≥60 years	167	62 (37.1)	2.77	1.539–4.972	0.001
**Smoking**					
No	704	182 (25.9)	1.00	-	-
Yes	141	39 (27.7)	1.10	0.731–1.645	0.656
**Alcohol Habits**					
No	782	201 (25.7)	1.00	-	-
Yes	62	19 (30.6)	1.28	0.727–2.243	0.395
**Ethnic Group**					
Ternatese	44	2 (4.5)	1.00	-	-
Aceh	73	14 (19.2)	4.98	1.075–23.091	0.040
Balinese	55	15 (27.3)	7.87	1.692–36.642	0.009
Batak	77	26 (33.8)	10.71	2.401–47.738	0.002
Bugis	83	25 (30.1)	9.05	2.032–40.319	0.004
Chinese	121	41 (33.9)	10.76	2.481–46.690	0.002
Dayak	45	17 (37.8)	12.75	2.730–59.538	0.001
Javanese	229	78 (34.1)	10.85	2.559–45.989	0.001
Malay	35	3 (8.6)	1.97	0.310–12.486	0.472
Minahasa	46	7 (15.2)	3.77	0.738–19.250	0.111
Nias	32	7 (21.9)	5.88	1.132–30.540	0.035
Kaili	12	2 (16.7)	4.20	0.526–33.540	0.176
Papuan	47	6 (12.8)	3.07	0.586–16.115	0.184
Timor	25	13 (52.0)	22.75	4.498–115.063	< 0.001
Tolaki	23	2 (8.7)	2.00	0.263–15.208	0.503

To analyze risk factors associated with *H*. *pylori*-negative gastritis we focused on samples from the Surabaya population (n = 224, including 99 Surabaya samples from our previous study [20]), which had detailed information regarding the demographics and lifestyle (including type of drinking water, type of toilet, washing hand habits, usage of PPIs, usage of antibiotics, usage of non-steroidal anti-inflammatory drugs (NSAIDs), usage of anti-anxiety medication, chili paste consumption, dried fish consumption, fresh fruits consumption, coconut milk consumption, and coffee and tea consumption habits). However, we found no association between those risk factors and the prevalence of *H*. *pylori*-negative gastritis.

### Atrophic gastritis and intestinal metaplasia

Overall, the prevalence of AG was 28.9% (305/1,053), which was higher in men than in women (P = 0.595, Table 1), whereas we observed 30 (2.8%) Indonesian dyspeptic patients had IM. Due to the small number of IM, we considered to combine the IM and AG into GC precursor lesion and calculating the risk factor. After combining into one classification, we observed men had higher prevalence of GC precursor lesion than women (Table 5). The age group showed a trend increasing age also increasing the odds of presence of GC prevalence but without any particular age group is significantly higher than the youngest age group. Balinese, Batak, Bugis, Dayak, Minahasanese, Papuan and Timor people had significantly higher odds of presence GC precursor lesion than Kaili and Tolaki people (OR, 5.24, 95%CI = 1.12–24.47; P = 0.035; OR, 9.04, 95%CI = 2.01–40.43; P = 0.004; OR, 6.56, 95%CI = 1.46–29.51; P = 0.014; OR, 5.16, 95%CI = 1.07–24.82; P = 0.041; OR, 6.67, 95%CI = 1.41–31.40; P = 0.016; OR, 11.34, 95%CI = 2.46–52.21; P = 0.002 and OR, 23.83, 95%CI = 4.81–118.10; P < 0.001, respectively, Table 5). Based on our previous reports, those ethnic groups had been reported to have a high prevalence of *H*. *pylori* infection [15, 16, 19], and indeed the *H*. *pylori*-infected patients showed considerably higher risks for presence of AG than non-infected patients (OR, 21.33; 95%CI, 11.922–38.178; P < 0.001); furthermore, after adjusting with the age, gender, and *H*. *pylori* infection, we observed Batak, Bugis, Dayak, Minahasa, Papuan and Timor ethnic group as independent risk factor for the presence of GC precursor lesion in dyspeptic patients in Indonesia (OR, 6.18, 95%CI = 1.22–31.19; P = 0.027; OR, 5.89, 95%CI = 1.16–29.82; P = 0.03, OR, 6.54, 95%CI = 1.21–35.1; P = 0.028, OR, 6.6, 95%CI = 1.24–35.04; P = 0.026; OR, 9.31, 95%CI = 1.78–48.69; P = 0.008; OR, 17.2, 95%CI = 3.06–96.94; P = 0.001).

**Table 5:** Risk factor of GC precusor in Indonesia.

Risk Factor	n	Case (%)	Crude OR	95%CI	P value
**Gender**					
Women	457	132 (28.9)	1.00	-	-
Men	596	183 (30.7)	1.09	0.835–1.424	0.523
**Age Group**					
≤ 18 y.o	6	1 (16.7)	1.00	-	-
18–29 y.o	118	26 (22.0)	1.41	0.158–12.635	0.757
30–39 y.o	226	54 (23.9)	1.57	0.179–13.730	0.684
40–49 y.o	257	77 (30.0)	2.14	0.246–18.613	0.491
50–59 y.o	250	88 (35.2)	2.72	0.312–23.614	0.365
≥ 60 y.o	189	68 (36.0)	2.81	0.322–24.548	0.350
**Smoking**					
no	784	230 (29.3)	1.00	-	-
yes	160	52 (32.5)	1.16	0.805–1.670	0.426
**Alcohol**					
no	867	253 (29.2)	1.00	-	-
yes	75	28 (37.3)	1.45	0.885–2.365	0.141
**Ethnic group**					
Tolaki	24	2 (8.3)	1.00	-	-
Kaili	12	1 (8.3)	1.00	-	-
Aceh	73	8 (11.0)	1.35	0.267–6.859	0.714
Balinese	62	20 (32.3)	5.24	1.121–24.477	0.035
Batak	102	46 (45.1)	9.04	2.019–40.437	0.004
Bugis	99	37 (37.4)	6.56	1.460–29.512	0.014
Chinese	128	30 (23.4)	3.37	0.749–15.146	0.114
Dayak	47	15 (31.9)	5.16	1.071–24.822	0.041
Javanese	233	52 (22.3)	3.16	0.720–13.875	0.127
Ternatese	47	12 (25.5)	3.77	0.770–18.467	0.101
Melayu	37	5 (13.5)	1.72	0.306–9.664	0.539
Minahasa	53	20 (37.7)	6.67	1.415–31.405	0.016
Nias	33	8 (24.2)	3.52	0.675–18.356	0.135
Papuan	65	33 (50.8)	11.34	2.465–52.202	0.002
Timor	38	26 (68.4)	23.83	4.810–118.101	<0.001
***H*. *pylori* status**					
Negative	947	223 (23.5)	1.00	-	-
Positive	106	92 (86.7)	21.33	11.922–38.178	<0.001

**Fig 2:**
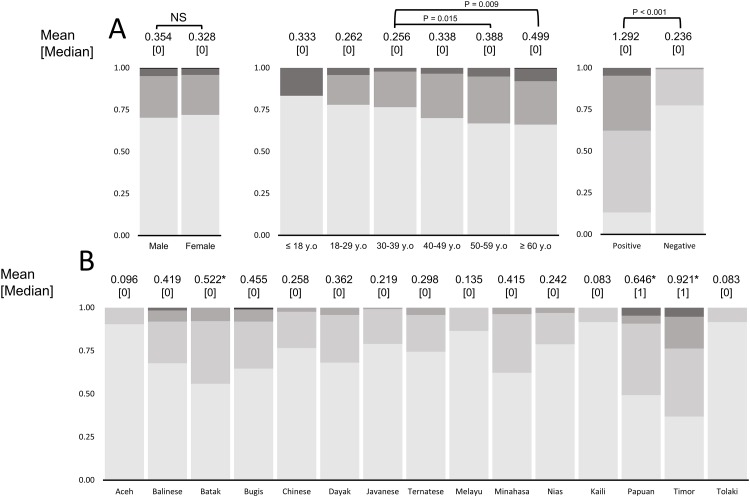
OLGA scores among Indonesian patients. (**A**) We observed the 50–59 and ≥60 year-old groups had significantly higher OLGA scores than those in the 30–39 age group. As expected the individuals infected with *H*. *pylori* had significantly higher OLGA scores than non-infected individuals. (**B**) We found three ethnic groups (Timor, Papuan, and Bataknese) with significantly higher OLGA scores than people in the Kaili ethnic group (P = 0.003, P = 0.025, and 0.040, respectively).

Based on the modified OLGA score, patients in both age groups (≥60 and between 50 and 59 years) showed the highest mean of OLGA score, and both were significantly higher than the patients between 30 and 39 years old (P = 0.009 and P = 0.015, respectively, Fig 2A). As expected, the people in the Timor ethnic group showed the highest mean OLGA score, followed by those in the Papuan and Bataknese ethnic groups. Moreover, those populations had significantly higher median scores than people in the Kaili ethnicity (mean[median] = 0.921[1], 0.646 [1] and 0.520 [0] vs. 0.083 [0], all P < 0.05, Fig 2B). After classifying the atrophic risk based on the OLGA scores as belonging to the high risk if the OLGA score >2 or to the low risk if the OLGA score ≤2 [25], the peoples in Timor and Papuan groups had the highest risk for gastric cancer based on the OLGA score (23.7% and 9.3%, respectively), and we found a significant association between ethnic group and the high-risk prevalence (P < 0.001) (S5 Table).

### Gastric cancer risk index

We analyzed gastric cancer risk using the modified GCRI [13]. The mean index score was higher in men than in women without a significant difference (mean [median] = 1.231 [1] and 1.190 [1], respectively, P = 0.132, Table 6). The highest GCRI index mean was that in the Timor ethnic group (1.684 [1]), followed by those in the Papuan, the Bugis, and the Minahasanese ethnic groups (1.492 [1], 1.404 [1], and 1.396 [1]); and we found significant differences in GCRI means among ethnic groups (P < 0.001). Those ethnic groups had higher risks and higher GCRI scores than the individuals in the rest of the population (OR, 3.19, 95%CI = 1.67–6.08; OR, 2.14, 95%CI = 1.30–3.51; OR, 1.57, 95%CI = 1.37–2.41; OR, 1.83, 95%CI = 1.06–3.17, respectively; all P < 0.05; S6 Table).

**Table 6:** The gastric cancer risk index score of Indonesian patients.

	n	Index Score								Mean[median]
		0	1	2	3	4	5	6	7	
**Gender**										
Male	596	56	395	112	26	4	2	0	1	1.231 [1]
Female	457	51	310	76	10	5	3	2	0	1.190 [1]
**Ethnic Group**										
Aceh	73	8	59	6	0	0	0	0	0	0.973 [1]
Balinese	62	4	44	11	2	0	1	0	0	1.242 [1]
Batak	102	8	61	26	5	2	0	0	0	1.333 [1]
Bugis	99	7	62	21	5	2	1	0	1	1.404 [1]
Chinese	128	22	85	16	4	0	1	0	0	1.047 [1]
Dayak	47	11	23	10	3	0	0	0	0	1.106 [1]
Javanese	233	30	167	31	4	1	0	0	0	1.052 [1]
Maluku	47	0	36	9	2	0	0	0	0	1.277 [1]
Melayu	37	0	33	3	0	0	0	1	0	1.216 [1]
Minahasa	53	3	32	13	4	1	0	0	0	1.396 [1]
Nias	33	2	24	6	1	0	0	0	0	1.182 [1]
Palu	12	2	9	1	0	0	0	0	0	0.917 [1]
Papuan	65	5	34	21	2	1	1	1	0	1.492 [1]
Timor	38	3	17	11	4	2	1	0	0	1.684 [1]
Tolaki	24	2	19	3	0	0	0	0	0	1.042 [1]

As the original usage of this index is for *H*. *pylori*-infected patients [13], we analyzed only the patients with *H*. *pylori* infections. Because several ethnic groups had very low numbers of infected patients, we only analyzed ethnic groups containing 5 or more infected patients; therefore, we only counted 7 ethnic groups for this particular analysis (S6 Table). Among those ethnic groups, we observed the Balinese and Minahasanese peoples had the highest GCRI mean scores (both 2.429 [2]), followed by the Timor and Papuan ethnic groups (2.308 [2.5] and 2.056 [2], respectively, S7 Table), but we did not observe any significant difference in GCRI index mean scores between the groups (P = 0.477).

In addition to our observations among *H*. *pylori*-infected patients, we also analyzed GCRI score among *H*. *pylori*-negative patients. People from Timor yielded the highest GCRI mean score (1.360 [1]) followed by Papuan (1.277 [1]), Bugis and Bataknese (1.277 [1]), and Minahasanese ethnic groups (1.239 [1]), and we observed significant differences in the GCRI mean scores among ethnic groups (P = 0.038). An ordinal regression model showed only Timor and Papuan had higher odds to have higher GCRI index value than Kaili (OR = 4.20, 95%CI = 1.15–18.53, P = 0.02 and OR = 3.94, 95%CI = 1.01–15.40, P = 0.04, respectively). In our multivariate analysis, after adjusting for age, gender, and *H*. *pylori* infection, we found that only the Minahasanese group had higher GCRI scores than the rest of the study population (OR, 1.69, 95%CI = 0.98–2.94, P = 0.067).

**Fig 3:**
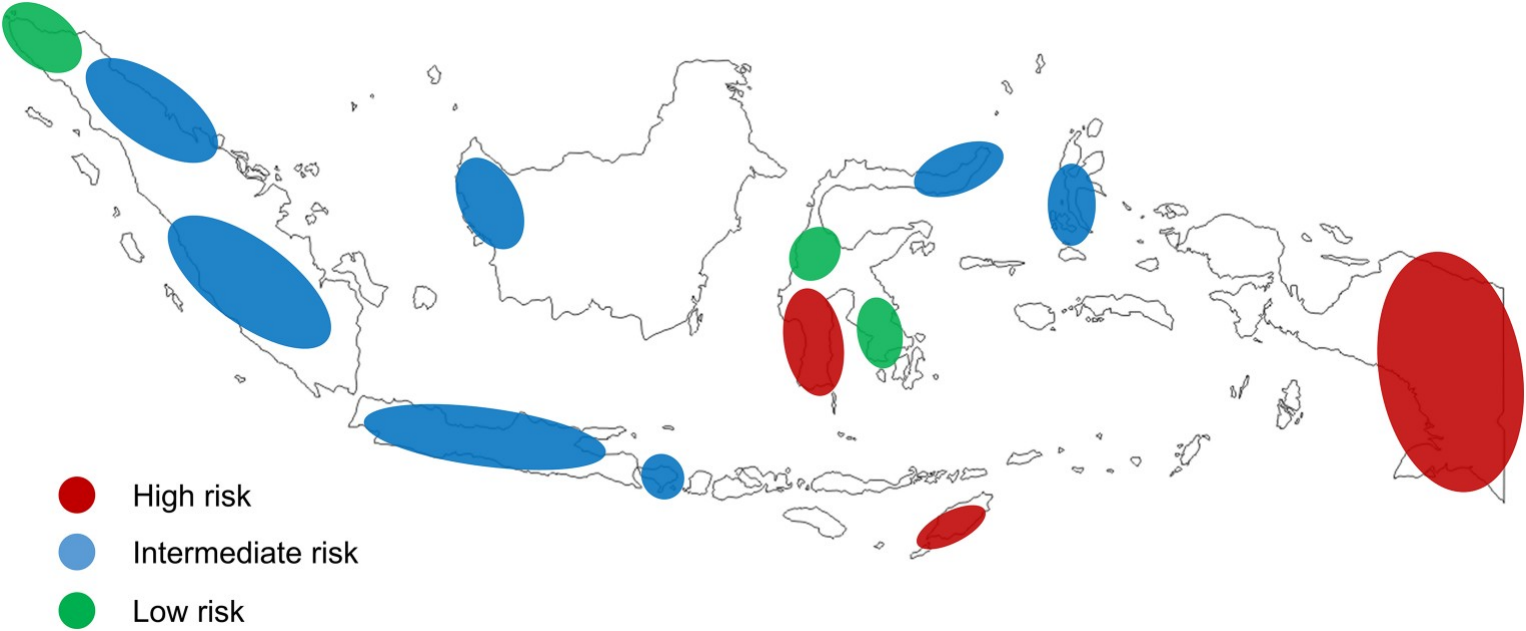
Map of gastric cancer risk among ethnic group in Indonesia.

Based on AG prevalence and GCRI scores we classified Indonesian ethnic groups as having high, intermediate, or low risks for gastric cancer development. Timor, Papuan, Buginese, and Bataknese ethnic groups were considered the most vulnerable ethnic groups to develop gastric cancer, whereas Tolaki and Aceh ethnic groups had have the least known risks for gastric cancer development. The other populations can be considered as having intermediate risks. Fig 3 shows a gastric cancer risk map among Indonesian populations.

## Discussion

We conducted a nationwide study to examine gastric mucosal status based on histological examination to determine gastric cancer risks among ethnic groups in Indonesia. We observed that almost half of the histological specimens had at least one type of gastric abnormality, which is considerably lower than those in reports in Egypt (65.7%) and Nigeria (71.7%) [34, 35]. However, this was predictable due to the higher prevalence of *H*. *pylori* infection in those countries compared to that in Indonesia [36]. In fact, dyspepsia is the sixth and fifth symptom of the 10 most prevalent outpatient and inpatient cases in Indonesia, respectively [37], suggesting gastritis is still present in many individuals of the Indonesian patients. Our results also showed that *H*. *pylori* infection had a great impact on the gastric mucosa changes including active gastritis, chronic gastritis, AG, and IM regardless of ethnic group variables. *H*. *pylori* itself is a strong independent risk factor for development of gastric cancer with or without the presence of AG [38]. We found that acute gastritis was higher in male and female. Our finding also similar to the finding from study in rats which active gastritis was higher in male rats than the female one. The higher active gastritis in male rats was attributed by decreasing of proliferation rates [39]. In addition, sex hormone was also had a critical role in the development of gastric pathology in the mice [40].

Overall, the prevalence of AG was considerably low, even compared to those in neighboring countries such as Malaysia (42.3% [41]), or Japan and China (52 to 82% [9, 42]). Indonesian GCRI scores were also considerably lower than those in Japanese people [13], even when we analyzed the *H*. *pylori*-positive patients only (2.047 vs. 4.071), supporting the fact that Japan has a higher gastric cancer incidence than Indonesia (ASR: 27.5/100,000 vs. 1.2/100,000; GLOBOCAN, 2018). However, there is no particular index threshold which can significantly determine the gastric cancer. Further study to determine significant index score is necessary. In addition, people in the Timor ethnic group had a higher prevalence of AG than Japanese people. In addition, half of the Timor ethnic population in our study had a high-risk score based on the OLGA classification. We also found a significant difference of mean distributions in ethnic groups, with the Timor, Papuan, and Buginese groups having the highest risk for developing gastric cancer in Indonesia, regardless of *H*. *pylori* infection prevalence. These data suggest the important role of ethnic group to the gastric cancer development. The different ethnic group may influence different gene polymorphism, food, diet and epigenetic interaction which had been reported to play a critical role in the cancer pathogenesis, including gastric cancer [43].

The location of predominant gastritis may affect the course of the disease. The antral-predominant gastritis type is more likely to become peptic ulcer diseases, whereas the corpus-predominant or pan-gastritis (with reduced acid secretion) is more likely to develop AG and gastric cancer [44]. Our topology analysis showed Indonesian patients had mostly the antral-predominant chronic gastritis, supporting the low risk for gastric cancer reported for Indonesia (this type is more likely to progress into peptic ulcer disease than into gastric cancer). In fact, the peptic ulcer prevalence in Indonesia is also lower than that in other countries such as India or Malaysia [45]. The comparison of two countries between Japan and the UK showed that antral-predominant gastritis results in a lower risk for gastric cancer development than corpus-predominant gastritis and pan-gastritis [46]. In addition, China has a high gastric cancer incidence country (ASR: 22.73/100.000), and its people showed more cases of high corporal-predominant gastritis [47] than of antral-predominant gastritis.

We observed ~27% of *H*. *pylori*-negative patients had gastritis in their stomach upon histological examination. This number is similar to that in the USA (between 18 and 21% [33, 48]). Our data showed women were more likely to develop *H*. *pylori*-negative gastritis than men. This result differs from an observation in veterans in the USA [48]. However, our results may reflect the population-based observation with an almost equal proportion of men and women; and indeed another study examining *H*. *pylori*-negative gastritis in a population-based study also showed the women were more likely to develop *H*. *pylori*-negative gastritis than men [49]. In addition, our result showed the older age groups, especially people in their 5^th^ and 6^th^ decades had a significantly higher risk to develop gastritis in the absence of *H*. *pylori*. Although the autoimmune background of *H*. *pylori*-negative gastritis was rare [48], the older age individuals had higher chances of developing autoimmune diseases, which may be associated to the presence of gastritis [50]. Our ethnic comparisons also showed that people in Timor, Dayak, Javanese, Chinese, and Bataknese populations had higher risks for developing *H*. *pylori*-negative gastritis even we have changed the ethnic group for comparison. The absence of Papuan and the appearance of Dayak and Javanese groups as the ethnicities at higher risk in this particular analysis suggests a novel still unknown risk factor and mechanism for developing *H*. *pylori*-negative gastritis. In addition, we tried to elucidate the risk factors with more detailed socio-demographic data in Surabaya, an area with low of *H*. *pylori* infection, but we could not find an association. Therefore, further studies on *H*. *pylori*-negative gastritis are necessary, considering that the rate of *H*. *pylori* infection in Indonesia was very low.

Increasing activity in the presence of chronic inflammation related to *H*. *pylori* infection may be another pathway promoting development of gastric carcinoma in the absence of AG [51]. *H*. *pylori* infection has been thought to alter several biological functions, including increasing oxidative stress, and together with intracellular and genetic and epigenetic alterations leads to gastric cancer [38, 52], including involvement of peptidyl prolyl cis, trans-isomerase of *H*. *pylori* (HP0175) that could influence gastric Th17 response, and finally could provide more clear association between *H*. *pylori* infection and development gastric cancer by promoting pro-inflammatory matrix degradation and pro-angiogenic pathways [53]. However, our results showed the prevalence of chronic active gastritis was associated with the prevalence of AG, suggesting that chronic AG may also develop into gastric carcinoma. This suggests that increasing the activity in chronic gastritis can lead to cancer development in the either presence or absence of AG manner, although a cohort study is required to prove it. Interestingly, our data also showed individuals with AG not associated with the IM prevalence. In the pathway of gastric carcinoma development, especially the intestinal type, the mucosal changes from AG to IM are influenced by many variables, including low acidity, CDX2 expression, D1S191 instability, and telomere reduction [38]. Our data showed the AG was antral-predominant (low number of parietal cells producing hydrochloric acid due to atrophic glands, resulting in hypochlorhydria) and support the idea that a low-acidity condition leads to IM in the presence of AG. However, the involvement of other genetic factors to accelerate IM development may be more important than the gastric condition alone. In this regard, further research, especially genetic involvement in IM development, in Indonesian population is needed.

Our study may explain the gastric mucosal changes in the population with low *H*. *pylori*. However, our current study population was only analyzed the dyspeptic patients, thus our finding only could be applied to dyspeptic patients. Furthermore, next study involving total population is necessary. Also, due to the considerably low sample number and low number of infected individuals, some analyses on *H*. *pylori*-infected individuals could only be performed with a partition of many ethnic groups, resulting in a low number of analyzed samples in each group. In addition, our use of the modified OLGA scoring system, instead of the OLGA scores (due to the different numbers of analyzed specimens), may reduce the reliability of the scoring system. Cancer disease are a complex development disease which involve interaction between host, agent and environmental factors. The environmental factor, such as diet and microbiome; the host factors such as gene polymorphism and interaction between host and the environmental factor such as epigenetic profile may play critical role to the development of gastritis as well as gastric cancer. Therefore, it is interesting to find out the role of them in the future study. The finding that the people in the Timor ethnic group have the highest risks for *H*. *pylori*-negative gastritis development suggests the Indonesian government should focus its prevention and intervention plans in this particular ethnic group with the highest risks for developing many gastroduodenal diseases, including, *H*. *pylori*-negative gastritis, AG and even gastric cancer.

## Conclusions

We showed that Indonesia is a low-risk gastric cancer country after analysis of our survey on gastric mucosal features in Indonesian patients with dyspepsia. However, several ethnic groups had severe gastric mucosa conditions characteristic of high-risk populations. Our results may be useful to plan screenings and interventions focused on the individuals in those ethnic groups with the highest risks for developing gastroduodenal diseases including gastric cancer.

## Supporting information

S1 Table The Gastric mucosal condition of Indonesian *H*. *pylori* negative patients. (DOCX)

S2 Table The prevalence of severe chronic gastritis among ethnic groups in Indonesia. (DOCX)

S3 Table The association between activity in the chronic gastritis and the atrophic gastritis and intestinal metaplasia. (DOCX)

S4 Table Prevalence of High risk based on OLGA score of Indonesian population. (DOCX)

S5 Table The odd ratio of the *H*. *pylori* negative gastritis prevalence in comparison to Tolaki ethnic. (DOCX)

S6 Table The Odd ratio developing higher GCRI index of Timor, Papuan, Bugis and Minahasnese compare to the rest of studied population. (DOCX)

S7 Table GCRI index score among *H*. *pylori* infected patients in Indonesia. (DOCX)
