# Supplementary material for: Analysis of risks of gastric cancer by gastric mucosa among Indonesian ethnic groups
Source: PLoS One. 2019 May 9;14(5):e0216670. doi: 10.1371/journal.pone.0216670 (PMC6508733; doi:10.1371/journal.pone.0216670)
Supplement: S1 Table — (DOCX) [file pone.0216670.s001.docx]

**S1 Table.** The Gastric Mucosal Condition of Indonesian *H. pylori* negative patients.

| **Characteristic** | **n** | **Type of Abnormality (%)** | | | |
| --- | --- | --- | --- | --- | --- |
|  |  | **Acute** | **Chronic** | **Atrophy** | **IM** |
| Male | 534 | 39 (7.3) | 126 (23.6) | 124(23.2) | 12 (2.2) |
| Female | 413 | 39 (9.4) | 119 (28.8) | 89(21.5) | 5 (1.2) |
| Ethnic Group |  |  |  |  |  |
| Aceh | 73 | 3 (4.1) | 12 (16.4) | 7 (9.6) | 1 (1.3) |
| Balinese | 55 | 5 (9.1) | 15 (27.3) | 14 (25.5) | 0 (0.0) |
| Batak | 77 | 10 (12.9) | 25 (32.4) | 23 (29.9) | 1 (1.3) |
| Bugis | 83 | 5 (6.0) | 23 (27.7) | 20 (24.1) | 4 (4.8) |
| Chinese | 121 | 10 (8.2) | 41 (33.9) | 24 (19.9) | 1 (0.8) |
| Dayak | 45 | 7 (15.5) | 17 (37.8) | 13 (28.9) | 1 (2.2) |
| Javanese | 229 | 19 (8.2) | 74 (32.3) | 48 (20.9) | 4 (1.7) |
| Ternatese | 44 | 1 (2.3) | 1 (2.3) | 9 (20.5) | 0 (0.0) |
| Melayu | 35 | 3 (8.6) | 1 (2.8) | 4 (11.4) | 0 (0.0) |
| Minahasa | 46 | 0 (0.0) | 7 (15.2) | 13 (28.3) | 1 (2.2) |
| Nias | 32 | 4 (12.5) | 6 (18.8) | 6 (18.8) | 1 (3.1) |
| Kaili | 12 | 1 (8.3) | 2 (16.7) | 1 (8.3) | 0 (0.0) |
| Papuan | 47 | 2 (4.2) | 6 (12.8) | 17 (36.2) | 1 (2.1) |
| Timor | 25 | 7 (28.0) | 13 (52.0) | 12 (48.0) | 2 (8.0) |
| Tolaki | 23 | 1 (4.3) | 2 (8.7) | 2 (8.7) | 0 (0.0) |
