# Supplementary material for: Analysis of risks of gastric cancer by gastric mucosa among Indonesian ethnic groups
Source: PLoS One. 2019 May 9;14(5):e0216670. doi: 10.1371/journal.pone.0216670 (PMC6508733; doi:10.1371/journal.pone.0216670)
Supplement: S2 Table — (DOCX) [file pone.0216670.s002.docx]

**S2 Table.** The Prevalence of Severe Chronic Gastritis Among Ethnic Groups in Indonesia

| **Ethnic Groups** | **n** | **Degree of Severity (%)** | | |
| --- | --- | --- | --- | --- |
|  |  | **Mild** | **Severe** | |
| Aceh | 12 | 12 (100.00) | 0 (0.00) | |
| Balinese | 22 | 20 (90.91) | 2 (9.09) | |
| Batak | 49 | 43 (87.76) | 6 (12.24) | |
| Bugis | 39 | 35 (89.74) | 4 (10.26) | |
| Chinese | 48 | 47 (97.92) | 1 (2.08) | |
| Dayak | 19 | 16 (84.21) | 3 (15.79) | |
| Javanese | 78 | 78 (100.00) | 0 (0.00) | |
| Ternatese | 4 | 4 (100.00) | 0 (0.00) | |
| Melayu | 3 | 3 (100.00) | 0 (0.00) | |
| Minahasa | 14 | 12 (85.71) | 2 (14.29) | |
| Nias | 7 | 7 (100.00) | 0 (0.00) | |
| Kaili | 2 | 2 (100.00) | 0 (0.00) | |
| Papuan | 24 | 20 (83.33) | 4 (16.67) | |
| Timor | 26 | 21 (80.77) | 5 (19.23) | |
| Tolaki | 3 | 3 (100.00) | 0 (0.00) |  |
