# Supplementary material for: Analysis of risks of gastric cancer by gastric mucosa among Indonesian ethnic groups
Source: PLoS One. 2019 May 9;14(5):e0216670. doi: 10.1371/journal.pone.0216670 (PMC6508733; doi:10.1371/journal.pone.0216670)
Supplement: S3 Table — (DOCX) [file pone.0216670.s003.docx]

**S3 Table.** **The association between activity in the chronic gastritis and the atrophic gastritis and intestinal metaplasia.**

| **Type of Chronic Gastritis** | **n** | **AG (%)** | **IM (%)** |
| --- | --- | --- | --- |
| **Total** | 350 |  |  |
| Acute chronic gastritis | 164 | 126 (76.8) | 12 (7.3) |
| Non-acute chronic gastritis | 186 | 54 (40.9) | 12 (6.5) |
