# Supplementary material for: Analysis of risks of gastric cancer by gastric mucosa among Indonesian ethnic groups
Source: PLoS One. 2019 May 9;14(5):e0216670. doi: 10.1371/journal.pone.0216670 (PMC6508733; doi:10.1371/journal.pone.0216670)
Supplement: S4 Table — (DOCX) [file pone.0216670.s004.docx]

**S4 Table. Prevalence of High risk based on OLGA score of Indonesian population.**

| **Ethnic Group** | **n** | **High Risk (%)** |
| --- | --- | --- |
| Aceh | 73 | 0 (0.0) |
| Balinese | 62 | 5 (8.1) |
| Batak | 102 | 8 (7.8) |
| Bugis | 99 | 8 (8.1) |
| Chinese | 128 | 3 (2.3) |
| Dayak | 47 | 2 (4.3) |
| Javanese | 233 | 2 (0.9) |
| Ternatese | 47 | 2 (4.3) |
| Melayu | 37 | 0 (0.0) |
| Minahasa | 53 | 2 (3.8) |
| Nias | 33 | 1 (3.0) |
| Kaili | 12 | 0 (0.0) |
| Papuan | 65 | 6 (9.2) |
| Timor | 38 | 9 (23.7) |
| Tolaki | 24 | 0 (0.0) |
