# Supplementary material for: Analysis of risks of gastric cancer by gastric mucosa among Indonesian ethnic groups
Source: PLoS One. 2019 May 9;14(5):e0216670. doi: 10.1371/journal.pone.0216670 (PMC6508733; doi:10.1371/journal.pone.0216670)
Supplement: S5 Table — (DOCX) [file pone.0216670.s005.docx]

**S5 Table. The odd ratio of the *H. pylori* negative gastritis prevalence in comparison to Tolaki ethnic.**

| **Ethnic Group** | **OR** | **95% CI** | **P value** |
| --- | --- | --- | --- |
| Tolaki | 1.000 | - | - |
| Maluku | 0.500 | 0.066 – 3.802 | 0.503 |
| Aceh | 2.492 | 0.522 – 11.893 | 0.252 |
| Balinese | 3.937 | 0.822 – 18.871 | 0.087* |
| Batak | 5.353 | 1.164 – 24.606 | **0.031** |
| Bugis | 4.526 | 0.986 – 20.783 | 0.052* |
| Chinese | 5.381 | 1.203 – 24.080 | **0.028** |
| Dayak | 6.375 | 1.325 – 30.661 | **0.021** |
| Javanese | 5.424 | 1.240 – 23.730 | **0.025** |
| Melayu | 0.984 | 0.151 – 6.399 | 0.987 |
| Minahasa | 1.885 | 0.359 – 9.898 | 0.454 |
| Nias | 2.940 | 0.551 – 15.698 | 0.207 |
| Palu | 2.100 | 0.257 – 17.143 | 0.489 |
| Papuan | 1.537 | 0.285 – 8.282 | 0.617 |
| Timor | 11.375 | 2.187 – 59.172 | **0.004** |
