# Supplementary material for: Analysis of risks of gastric cancer by gastric mucosa among Indonesian ethnic groups
Source: PLoS One. 2019 May 9;14(5):e0216670. doi: 10.1371/journal.pone.0216670 (PMC6508733; doi:10.1371/journal.pone.0216670)
Supplement: S6 Table — (DOCX) [file pone.0216670.s006.docx]

**S6 Table. The Odd ratio developing higher GCRI index of Timor, Papuan, Bugis and Minahasnese compare to the rest of studied population.**

| **Ethnic Groups** | **OR** | **95% CI** | **P value** |
| --- | --- | --- | --- |
| Timor | 3.19 | 1.677 – 6.080 | < 0.001 |
| Papuan | 2.14 | 1.303 – 3.515 | 0.002 |
| Bugis | 1.57 | 1.036 – 2.409 | 0.035 |
| Minahasanese | 1.83 | 1.058 – 3.168 | 0.032 |
