# Supplementary material for: Analysis of risks of gastric cancer by gastric mucosa among Indonesian ethnic groups
Source: PLoS One. 2019 May 9;14(5):e0216670. doi: 10.1371/journal.pone.0216670 (PMC6508733; doi:10.1371/journal.pone.0216670)
Supplement: S7 Table — (DOCX) [file pone.0216670.s007.docx]

**S7 Table. GCRI index score among *H. pylori* infected patients in Indonesia.**

| **Ethnic Groups** | **n** | **GCRI index Score** | | | | | | | **Mean (median)** |
| --- | --- | --- | --- | --- | --- | --- | --- | --- | --- |
|  |  | **0** | **1** | **2** | **3** | **4** | **5** | **6** |  |
| Balinese | 7 | 0 | 2 | 2 | 2 | 0 | 1 | 0 | 2.429 (2) |
| Batak | 25 | 0 | 10 | 10 | 4 | 1 | 0 | 0 | 1.840 (2) |
| Bugis | 16 | 0 | 5 | 7 | 3 | 0 | 1 | 0 | 2.063 (2) |
| Chinese | 7 | 0 | 5 | 0 | 1 | 0 | 1 | 0 | 1.857 (1) |
| Minahasa | 7 | 0 | 0 | 4 | 3 | 0 | 0 | 0 | 2.429 (2) |
| Papuan | 18 | 1 | 7 | 6 | 1 | 1 | 1 | 1 | 2.056 (2) |
| Timor | 13 | 1 | 2 | 6 | 1 | 2 | 1 | 0 | 2.308 (2) |
